# Anti-inflammatory, gastroprotective and anti-ulcerogenic effects of red algae *Gracilaria changii* (Gracilariales, Rhodophyta) extract

**DOI:** 10.1186/1472-6882-13-61

**Published:** 2013-03-14

**Authors:** Meng-Hooi Shu, David Appleton, Keivan Zandi, Sazaly AbuBakar

**Affiliations:** 1 Tropical Infectious Diseases Research and Education Center (TIDREC), Department of Medical Microbiology, Faculty of Medicine, University of Malaya, Kuala Lumpur 50603, Malaysia; 2 Sime Darby Technology Centre, 1st Floor, Block B UPM-MTDC Technology Centre III, Lebuh Silikon, Universiti Putra Malaysia, Serdang, Selangor 43400, Malaysia

**Keywords:** Anti-inflammatory, Anti-ulcerogenic, Gastroprotective, *Gracilaria changii*, Cytokines, Betamethasone, Omeprazole, Seaweeds, Ulcer

## Abstract

**Background:**

*Gracilaria changii* (Xia et Abbott) Abbott, Zhang et Xia, a red algae commonly found in the coastal areas of Malaysia is traditionally used for foods and for the treatment of various ailments including inflammation and gastric ailments. The aim of the study was to investigate anti-inflammatory, gastroprotective and anti-ulcerogenic activities of a mass spectrometry standardized methanolic extract of *Gracilaria changii*.

**Methods:**

Methanolic extract of *Gracilaria changii* (MeOHGCM6 extract) was prepared and standardized using mass spectrometry (MS). Anti-inflammatory activities of MeOHGCM6 extract were examined by treating U937 cells during its differentiation with 10 μg/ml MeOHGCM6 extract. Tumour necrosis factors-α (TNF-α) response level and TNF-α and interleukin-6 (IL-6) gene expression were monitored and compared to that treated by 10 nM betamethasone, an anti-inflammatory drug. Gastroprotective and anti-ulcerogenic activities of MeOHGCM6 extract were examined by feeding rats with MeOHGCM6 extract ranging from 2.5 to 500 mg/kg body weight (b.w.) following induction of gastric lesions. Production of mucus and gastric juice, pH of the gastric juice and non-protein sulfhydryls (NP-SH) levels were determined and compared to that fed by 20 mg/kg b.w. omeprazole (OMP), a known anti-ulcer drug.

**Results:**

MS/MS analysis of the MeOHGCM6 extracts revealed the presence of methyl 10-hydroxyphaeophorbide *a* and 10-hydroxypheophytin *a*, known chlorophyll proteins and several unidentified molecules. Treatment with 10 μg/ml MeOHGCM6 extract during differentiation of U937 cells significantly inhibited TNF-α response level and TNF-α and IL-6 gene expression. The inhibitory effect was comparable to that of betamethasone. No cytotoxic effects were recorded for cells treated with the 10 μg/ml MeOHGCM6 extract. Rats fed with MeOHGCM6 extract at 500 mg/kg b.w. showed reduced absolute ethanol-induced gastric lesion sizes by > 99% (*p* < 0.05). This protective effect was comparable to that conferred by OMP. The pH of the gastric mucus decreased in dose-dependent manner from 5.51 to 3.82 and there was a significant increase in NP-SH concentrations.

**Conclusions:**

Results from the study, suggest that the mass spectrometry standardized methanolic extract of *Gracillaria changii* possesses anti-inflammatory, gastroprotective and anti-ulcerogenic properties. Further examination of the active constituent of the extract and its mechanism of action is warranted in the future.

## Background

Inflammation is a major feature of many diseases. It is characterized by a complex of orchestrated interactions between mediators of inflammation and inflammatory cells directed toward removing irritants and healing of tissue injuries [1,2]. Inflammation is an important host defence mechanism. Inflammation that occurs in the mucosal of gastrointestinal tract, however, causes gastrointestinal ulcer [3]. Gastrointestinal ulcer is a common major disorder of the digestive system affecting millions of Americans and many more worldwide [4]. The most common cause of gastrointestinal ulcer disease are infection with *Helicobacter pylori* (*H. pylori*) [5] and long-term use of nonsteroidal anti-inflammatory drugs (NSAIDs) such as aspirin and ibuprofen [6]. The advent of antibiotic controls against *H. pylori* has significantly reduced the number of gastrointestinal ulcer cases in the developed countries [7]. The disease however, is still high in other countries and the disease caused by the use of NSAIDs is still a major health concern in the developed countries [6,7].

Treatment for inflammation in general, is aimed at either inhibiting the activity of inflammatory cells or inhibiting the production of inflammatory mediators [8]. The later can be accomplished using drugs such as NSAIDs and corticosteroids [9]. Treatment for gastrointestinal ulcer in general, is aimed at either eliminating *H. pylori* infection or reducing and inhibiting the levels of gastric acid production responsible for the erosion of gastrointestinal protective layer [10,11]. The later can be accomplished using drugs such as histamine (H_2_)-blockers, acid pump inhibitors and mucosal protective medications [9]. However, various side effects of long term use of these drugs have been described [9]. In addition, anti-inflammation and anti-ulcerogenic drugs are used mainly to alleviate the symptoms of the disease without actually treating or preventing the inflammatory and ulcerogenic processes.

Development of anti-inflammatory and anti-ulcerogenic drugs has recently focused on discovering the favourable application of herbal plant-derived extracts that are potent and safer to use. Algae found growing in abundance off the coastal areas of many parts of the world represent a huge yet untapped potential for new source of therapeutics [12,13]. The red algae for example has been reported to contain active compounds that may help ameliorate inflammation of the alimentary tract [14], prevent or treat gastric ulcers and cancers caused by oxidative stress [15,16], inhibit inflammatory activities by suppressing the production of inflammatory mediators [17-19] and induce cancer cell apoptosis in stomach [20] and colon [21]. Natural compounds derived from the edible algae could be safer to be used as anti-inflammatory and gastric anti-ulcerogenic therapeutics as they have been taken as food and used in traditional medicines since time immemorial [13].

The red algae, *Gracilaria changii* (Xia et Abbott) Abbott, Zhang et Xia found growing off the coastal areas of Malaysia represents a potential local source of this naturally-derived therapeutics. Presently, the algae are commercially harvested only for its colloidal agar content and as local food delicacies. These algae are widely applied as folk medicine for the treatment of various ailments including inflammation and gastric ailments. The present study aims to evaluate the potential anti-inflammatory, gastroprotective and anti-ulcerogenic activities of these Malaysian red algae, *Gracilaria changii*.

## Methods

### Algae extract preparation

*Gracilaria changii* (Xia et Abbott) Abbott, Zhang et Xia were collected from the coastal water of Morib, Selangor, Malaysia (N2° 44′ 908″, E101° 26′ 590″) in late February 2003 [22-24]. The algae were identified by Professor Dr. Phang Siew Moi, Director of the Institute of Ocean and Earth Sciences, Institute of Biological Sciences, Faculty of Science, University of Malaya, where the voucher specimens were maintained (herbarium no. PSM 6320 and PSM 6321). The algae were washed in sterile seawater with a final rinse in sterile distilled water as previously described [22]. The algae were then sonicated in a water bath sonicator and kept at −70°C. Methanolic extract of *Gracilaria changii* (MeOHGCM6 extract) was prepared at the University of Malaysia Terengganu (UMT) in accordance to the methods described [25]. The MeOHGCM6 extract was kept at −20°C prior to use.

### MeOHGCM6 extract

Profile analysis of the MeOHGCM6 extract was performed on a Shimadzu ultra-fast liquid chromatography (UFLC) system equipped with photodiode array ultraviolet (PDA UV) detector and ion trap time of flight (TOF) mass spectrometer (Shimadzu, Japan). The separation was achieved on a Waters Xbridge C18 column (50 mm × 2.1 mm, particle diameter of 2.5 μm) (Water, USA) at 40°C. The mobile phase consisted of water [0.1% formic acid (BDH Laboratory Supplies, England)] : acetonitrile (BDH Laboratory Supplies, England) (0.1% formic acid) at a flow rate of 0.50 ml/min with an injection volume of 10 μl. Electrospray ionization (ESI) mode with positive/negative switching and external reference was used for mass spectrometry (MS). The Dictionary of Natural Products (CRC Press) with high resolution MS spectra and structural information was used to analyse the positive and negative ion spectra along with MS/MS information for identification of similar components.

### Human promonocytic cell line (U937 cells)

U937 cells were purchased from American Type Culture Collection (ATTC, USA) and cultured in Roswell Park Memorial Institute-1640 (RPMI-1640 medium) (Flowlab, Australia) [supplemented with 1% of 10 mM non-essential amino acid (NEAA) (Flowlab, Australia) and 1% of 10 mM L-glutamine (L-Glu) (Flowlab, Australia)] containing 10% heat-inactivated fetal bovine serum (FBS) (Flowlab, Australia). Cells were incubated at 37°C in a humidified incubator with 5% CO_2_.

### Differentiation of U937 cells

U937 cells were seeded in 96-well cell culture plates (Falcon, USA) at a density of 2 × 10^4^ cells/well with RPMI-1640 medium containing 2% FBS and kept at 37°C in a humidified incubator with 5% CO_2_. The following day, medium containing phorbol 12-myristate 13-acetate (PMA) (Sigma-Aldrich, USA) at concentrations ranging from 0–50 nM were added to the U937 monocytic cells to induce differentiation for 24 and 48 hours. Medium containing only PMA diluents [dimethyl sulfoxide (DMSO) solution (Sigma-Aldrich, USA)] were added in parallel and used as vehicle control. At the end of the indicated time point, cells were evaluated for differentiation-specific macrophage cell surface marker expression and cell viability.

Expression of the CD11b, differentiation-specific macrophage cell surface marker was determined by staining cells as previously described [26,27]. Cell viability was determined using the trypan blue dye (Sigma, USA) exclusion assay as previously described [28].

### Cytotoxicity assay

U937 cells were seeded in 96-well cell culture plates at a density of 2 × 10^4^ cells/well with RPMI-1640 medium containing 2% FBS and kept at 37°C in a humidified incubator with 5% CO_2_. The following day, medium containing MeOHGCM6 extract (at concentrations ranging from 0–100 μg/ml) and betamethasone (Sigma, USA) (at concentrations ranging from 0–100 nM) were added. Medium containing only the MeOHGCM6 extract and betamethasone diluents [ethanol (EtOH) (BDH Laboratory Supplies, England) and DMSO, respectively] were added in parallel and used as vehicle control. A day post-treatment, the U937 cells proliferation rate was determined using the CellTiter 96® Non-Radioactive Cell Proliferation kit (Promega, USA) strictly following the manufacturer’s recommended protocol.

### MeOHGCM6 extract treatment of U937 cells

U937 cells were seeded in 24-well cell culture plates (Falcon, USA) at a density of 2 × 10^5^ cells/well with RPMI-1640 medium containing 2% FBS and incubated at 37°C in a humidified incubator with 5% CO_2_. The following day, the U937 cells were induced to differentiate with 10 nM PMA for 24 hours. During differentiation, the U937 cells were treated with the MeOHGCM6 extract or betamethasone at physiologic concentration. At 0, 8, 16 and 24 hours post-treatment, supernatant and cells were harvested and assayed for TNF-α response level and TNF-α and IL-6 gene expression.

### TNF-α response level

TNF-α level was measured using the BD OptEIA™ Human TNF (TNF-α) ELISA kit II (BD Biosciences, USA) according to the manufacturer’s protocol.

### TNF-α and IL-6 gene expression

Total RNA from the cell cultures were isolated using the TRI Reagent® (Molecular Research Centre, Inc., USA) and treated with DNase (Promega, USA). RNA was reverse-transcribed (cDNA) using the SuperScript™ II Reverse Transcriptase (Invitrogen, USA). The cDNA was then used to perform quantitative real-time PCR using SYBR® Green PCR master mix (Qiagen, Germany) on DNA Engine Opticon II® (MJ Research, Bio-Rad, USA) as previously described [29]. The oligonucleotide primers used were forward 5′AAGAGTTCCCCAGGGACCTC and reverse 5′GCTTGAGGGTTTGCTACAAC for TNF-α; forward 5′GAAAGGAGACATGTAACAAG and reverse 5′CCAGGCAAGTCTCCTCATTG for IL-6; and forward 5′GCGAGAAGATGACCCAGATC and reverse 5′GGATAGCACAGCCTGGATAG for beta-actin (β-actin) (internal reference).

### Animals

Adult female Sprague–Dawley rats aged between 7 to 8 weeks and weighing 160–200 g were used for the study. The protocol for animal experiments was approved by the Institutional Committee for Ethics in Animal Experimentation of the University of Malaya [Approval Number: MP/08/06/2010/SMH(R)]. The animals were obtained from University Putra Malaysia (UPM). The animals were housed individually in cages with wide-mesh wire bottoms to prevent coprophagy. They were kept in a temperature-controlled room that was well ventilated, with food and water, on a 12 hour light/dark cycle throughout the study. All rats were deprived of food for 48 hours, but had free access to drinking water until 2 hours prior to subjecting them to the ulcerogens.

### EtOH-induced gastric lesions

The rats were randomly divided into 7 groups with each group comprising of 6 rats. Group 1 was administered with 10% Tween 20 (extract diluents) (BDH Laboratory Supplies, England) and served as negative control group. Group 2 was given 500 mg/kg b.w. of the MeOHGCM6 extract-equivalent unrelated plant extract similarly prepared in parallel as MeOHGCM6 extract to serve as the non-specific plant extract (NSPE) control group. Group 3 was treated with OMP (Chemical Company of Malaysia, Malaysia) at 20 mg/kg b.w. and served as the positive treatment control group. The remaining group 4, 5, 6 and 7 received 4 different concentrations of the MeOHGCM6 extract ranging from 2.5 - 500 mg/kg b.w., respectively. After 30 minutes of pre-treatment, all animals were administered with absolute EtOH through the oral route. One hour post-administration, the animals were sacrificed by over-dosing them with diethyl ether (BDH Chemicals Ltd., England). The rat’s abdomen was dissected and the oesophagus nearest to the cardia and the distended stomach on the pyloric sphincter was immediately tied in a knot using a string to avoid leakage of the gastric contents. The stomach was rapidly removed and immersed in water.

### Measurement of gastric secretion

The gastric content of each rat stomach was aspirated and gently scraped using a spatula. The stomach juice containing food particles was discarded. The harvested gastric mucus was weighed using electronic balance (Mettler Toledo, Switzerland). The amount of gastric-juice was measured using a measuring cylinder. The pH of the gastric content was measured using a digital pH meter (Crison Instruments, S.A., Spain).

### Evaluation of gross gastric lesions

Following the harvest, the stomach was immediately flushed with saline and examined under a dissecting microscope (1.8 ×) with a square grid eyepiece. The glandular portion of the stomach was assessed for the formation of ulcer. The total ulcerating area (UA) of the haemorrhagic lesions for each stomach was measured by plannimetry (mm^2^) and the percentage of inhibition was calculated using the following formula:

$$\begin{array}{c} \text{Inhibition}\;\left(\%\right)=\left[\left(\mathrm{UA}\;\text{control}–\mathrm{UA}\;\text{treated}\right)/\mathrm{UA}\;\text{control}\right]\\ \;\times \;100 \end{array}$$

### Determination of production of non-protein sulfhydryls (NP-SH)

Gastric mucosal NP-SH level was measured as previously described [30] with minor modification. Briefly, the glandular stomach was weighed and homogenized in tubes containing ice-cold 0.02 M ethylenediaminetetraacetic acid (EDTA) (GibcoBRL Life Technologies, USA) (pH 8.9). Aliquots of the homogenates were mixed with distilled water and 50% trichloroacetic acid (Sigma Chemical Co., USA). The mixture was incubated with constant agitation for 10 minutes at 4°C and then centrifuged at 3000 × g for 15 minutes. The mixture was then assayed for NP-SH level using a glutathione assay kit (Sigma Chemical Co., USA) according to the manufacturer’s protocol.

### Statistical analysis

All data were expressed as mean ± standard deviation (S.D.) or median (25% and 75% quartile) from two or three independent experiments for anti-inflammatory studies and seven independent experiments for gastroprotective and anti-ulcerogenic studies. ANOVA (one-way analysis of variance and two-way analysis of variance) was used for the comparison among groups and for reporting the statistical significance ‘*p*’ with respect to the control group. The value of *p* < 0.05 was considered statistically significant. All statistical analyses were done using the GraphPad Prism 4.0 Software (USA) and SPSS version 16.0 Software (SPSS Inc, Chicago), respectively.

## Results

### Standardised MeOHGCM6 extract profile

**Figure 1:**
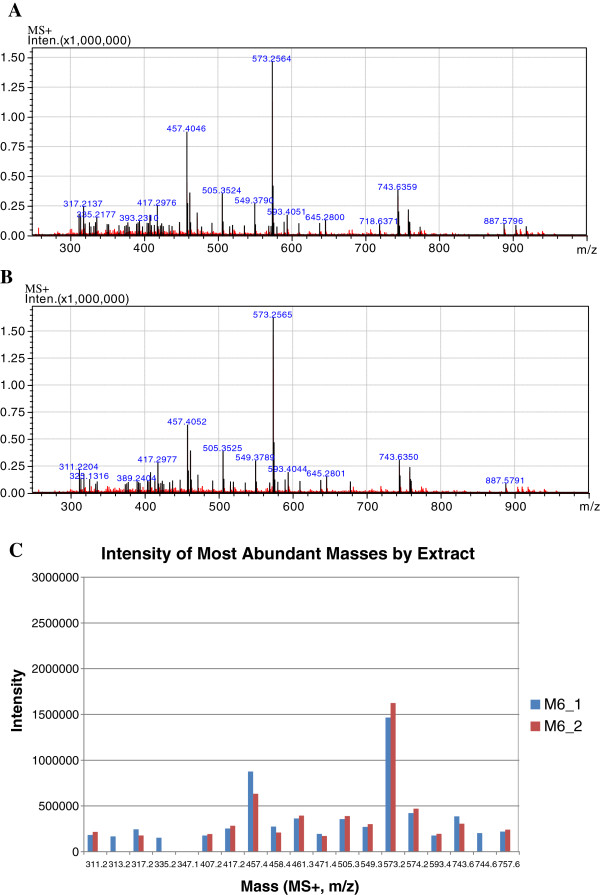
**Profiling of the different batches of MeOHGCM6 extract.** ESI mode with positive/negative switching and external reference was used for the MS using a Shimadzu UFLC system. Total positive ion spectra analysed for identification of similar components from the two different batches of the MeOHGCM6 extract used in the study. (**A**) MeOHGCM6 extract (Batch 1; M6-1). (**B**) MeOHGCM6 extract (Batch 2; M6-2). (**C**) Comparison of the intensity of the most abundant masses of the two MeOHGCM6 extract batches used in the study.

LC/MS analyses of the different batches of MeOHGCM6 extract were performed to ensure reproducibility and consistency of the extraction methods. Batch 1 and 2 of MeOHGCM6 extract used in the study showed highly similar mass spectral profiles and quantity of the major masses (Figure 1A-C). Analysis of the spectra and comparison to the Dictionary of Natural Products, CRC Press identified the distinctive masses as 457.405, 645.280 and 887.579. The identified mass in the positive ion spectrum of m/z 457.405, had fragments of 398.327 (base peak), 380.316 and 158.082. The neutral loss of 59.077 mass was possibly indicative of lost of trimethylamine. This suggests that the compound may contain a trimethylammonium moiety. The high resolution MS and MS/MS data could not definitively identify any known compounds from the Dictionary of Natural Products. The masses of m/z 645 and 887, however, showed a distinct association to two known chlorophylls, methyl 10-hydroxyphaeophorbide *a* and 10-Hydroxypheophytin *a* with the appropriate UV absorbances observed in the PDA analysis (Table 1).

**Table 1:** Structure identifications of the most significant mass present in MeOHGCM6 extract using LC/MS

**Mass (ion)**	**Found in batch**	**Possible ID**
417.298	1, 2	Most likely contaminant
457.405*	1, 2	Unidentified
461.326	1, 2	Most likely contaminant
505.353	1, 2	Most likely contaminant
573.256	1, 2	Unidentified
645.280	1, 2	Methyl 10-hydroxyphaeophorbide *a*
743.636	1, 2	Unidentified
887.579	1, 2	10-Hydroxypheophytin *a*

Dictionary of Natural Products, CRC Press with high resolution MS spectra were used to analyse the total positive ion spectra for the identification of possible chemical constituents.

* MS/MS (relative intensities%) 457.405 → 398.327 (100%), 380.316 (50%), 158.082 (40%).

### Differentiation-specific macrophage cell surface marker expression on U937 cells

**Figure 2:**
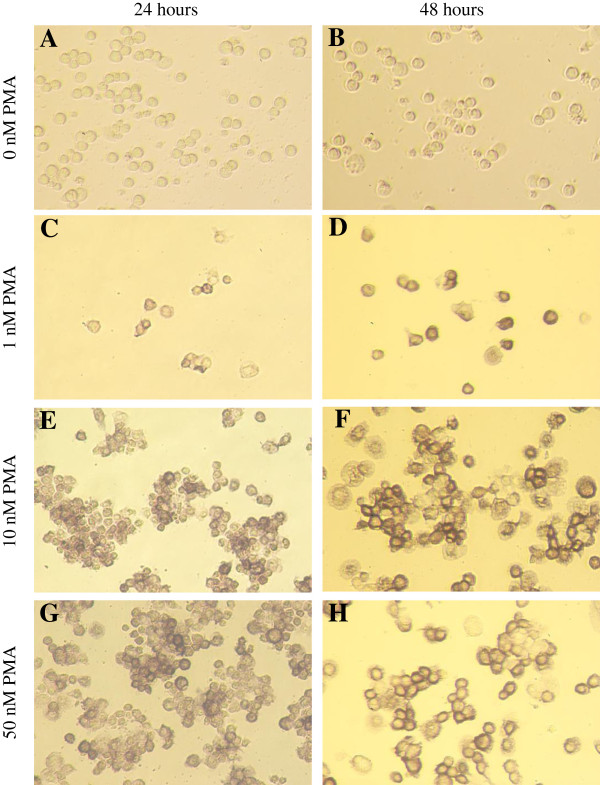
**Expression of the differentiation-specific macrophage cell surface marker on differentiated U937 monocytic cells.** U937 monocytic cells were induced to differentiate with (**A**) 0 nM PMA for 24 hours; (**B**) 0 nM PMA for 48 hours; (**C**) 1 nM PMA for 24 hours; (**D**) 1 nM PMA for 48 hours; (**E**) 10 nM PMA for 24 hours; (**F**) 10 nM PMA for 48 hours; (**G**) 50 nM PMA for 24 hours and (**H**) 50 nM PMA for 48 hours. Cells were stained using immunohistochemical staining to identify the presence of CD11b, a macrophage cell surface marker. Differentiated U937 monocytic cells were identified by the intense brownish blue stain on the cell surface of the differentiated cells. Cells were viewed at 20× magnifications under brightfield using an inverted microscope and photographed using a Nikon D70 camera (Nikon, Japan). One archetypal set of photographs from three separate experiments are shown.

**Figure 3:**
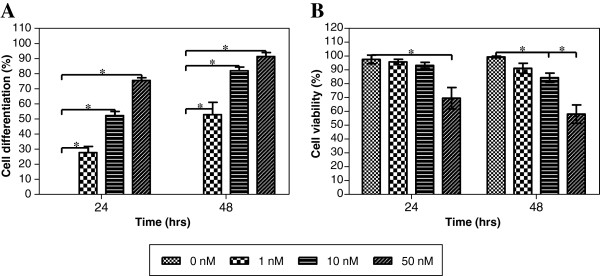
**Differentiation-specific U937 monocytic cells macrophage cell surface marker expression and cell viability determination.** U937 monocytic cells were induced to differentiate with various concentrations of PMA for 24 and 48 hours. (**A**) The percentages of differentiated cells were scored by the presence of CD11b on the cell surface. (**B**) The percentages of viable cells were scored using trypan blue exclusion assay. The percentage of differentiated or viable cells per total cells is the same prior to the experiment using the different concentrations of PMA and incubation period. Data are presented as mean ± S.D. from three independent experiments with ‘*’, *p* < 0.05 represent significance difference in comparison to the group that were not induced to differentiate.

Differentiated U937 cells are distinguished by the expression of the monocyte/macrophages lineage-specific CD11b, surface marker protein [26]. In our study, the expression of the CD11b was marked with an intense brownish blue labelling on the cell surface [27]. The expression of the CD11b was greatly increased when the U937 cells were induced to differentiate with increasing concentration of PMA (1, 10 and 50 nM) (Figure 2, Figure 3A). The finding suggested that treatment with 10 or 50 nM PMA for 24 hour or 1 nM PMA for 48 hour, differentiated U937 cells was well above 50% of the cell culture.

### Cell viability determination on differentiated U937 cells

The viable differentiated U937 cells were scored using the trypan blue dye exclusion assay. U937 cells viability exhibited a significant dose-dependent reduction in the percentage of viable cells when induced to differentiate with 1, 10 and 50 nM PMA (Figure 3B). The finding suggested that 90% of the differentiated U937 cells were still viable following treatment with 1 or 10 nM PMA for 24 hour or 1 nM PMA for 48 hour.

### Cytotoxicity of MeOHGCM6 extract on U937 cells

**Figure 4:**
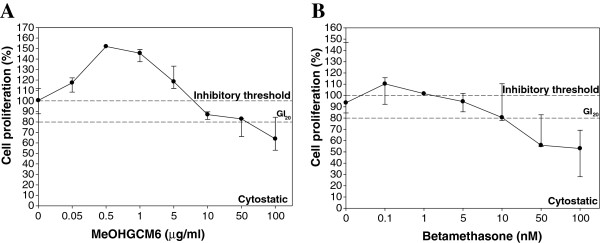
**Cytotoxicity effects of the various concentrations of MeOHGCM6 extract and betamethasone on U937 monocytic cells.** The proliferation rate of U937 monocytic cells treated with increasing concentrations of (**A**) MeOHGCM6 extract and (**B**) betamethasone was determined over a period of 24 hours by performing MTT assay. The percentage of cell proliferation is relative to the number of cells seeded prior to administrating the different doses of MeOHGCM6 extract and betamethasone. The concentrations of MeOHGCM6 extract that caused 30% (G1_30_), 50% (G1_50_) and 70% (G1_70_) of cell proliferation inhibition were extrapolated from the plot (indicated by an arrow). Results were expressed as the median (25% and 75% quartile) from two independent experiments.

The cytotoxic effects of the MeOHGCM6 extract and betamethasone on the U937 cells were determined by calculating the cell proliferation rate. Treated U937 cells with the MeOHGCM6 extract at 0.05, 0.5, 1 and 5 μg/ml exhibited proliferative bursts of 117.39% (108.48%, 122.10%), 152.04% (149.36%, 154.72%) (*p* < 0.05), 145.60% (137.48%, 149.11%) (*p* < 0.05) and 118.49% (112.01%, 133.28%), respectively (Figure 4A). Treatment with MeOHGCM6 extract at 10 μg/ml reduced the cell proliferation rate to 87.00% (82.43%, 89.62%). Treated U937 cells with the betamethasone exhibited significant dose-dependent reduction in their proliferation rate (Figure 4B). At lower concentrations of betamethasone (0.1, 1, 5 and 10 nM), the cell proliferation rate was 110.35% (92.20%, 115.83%), 113.75% (89.56%, 122.98%), 94.59% (85.56%, 101.93%) and 80.48% (77.78%, 110.48%), respectively.

### Effects of MeOHGCM6 extract treatment on the TNF-α response level during differentiation of the U937 cells

**Figure 5:**
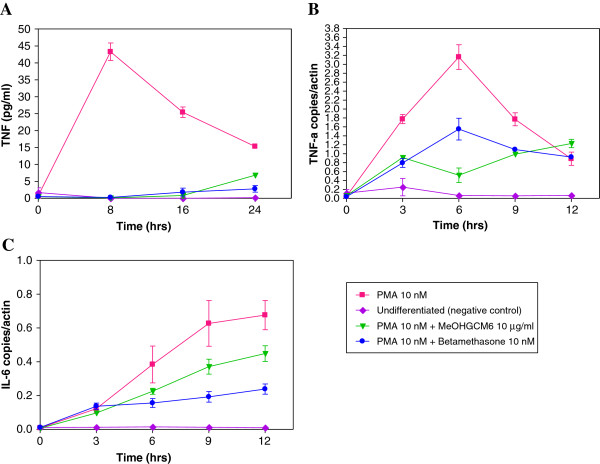
**MeOHGCM6 extract treatment on TNF-α response level and TNF-α and IL-6 gene expression during differentiation of the U937 monocytic cells.** The U937 monocytic cells were induced to differentiate with 10 nM PMA. During differentiation, U937 monocytic cells were treated with MeOHGCM6 extract. (**A**) The TNF-α response level were quantified using ELISA. The (**B**) TNF-α and (**C**) IL-6 gene expression levels were quantified using qRT-PCR. Results were expressed as the mean ± S.D. from quadruplicate assays.

The effects of MeOHGCM6 extract treatment on the regulation of TNF-α response level during the U937 cells differentiation was investigated using ELISA. The TNF-α response level reached a maximum level of 43.28 ± 5.15 pg/ml at 8 hours when the U937 cells were induced to differentiate (Figure 5A). In the presence of 10 μg/ml MeOHGCM6 extract during differentiation of U937 cells, the TNF-α response level significantly decreased till 8 hours [0.14 ± 0.28 pg/ml (*p* < 0.001)] and was comparable to that observed in the presence of betamethasone [0.28 ± 0.56 pg/ml (*p* < 0.001)].

### Effects of MeOHGCM6 extract treatment on the TNF-α and IL-6 gene expression during differentiation of the U937 cells

The regulation of TNF-α and IL-6 by MeOHGCM6 extract treatment during the U937 cells differentiation was further investigated at the gene expression level using qRT-PCR. The TNF-α gene expression level peaked after 6 hours (3.16 ± 0.48 copies/actin) of the U937 cells differentiation (Figure 5B). TNF-α gene expression level decreased during the first 6 hours [0.52 ± 0.28 copies/actin (*p* < 0.001)] of U937 cells differentiation in the presence of 10 μg/ml MeOHGCM6 extract. The decreased in the TNF-α gene expression level of U937 cells during differentiation when treated with 10 μg/ml MeOHGCM6 was comparable to or better than the findings from the treatment with 10 nM betamethasone [0.55 ± 0.42 copies/actin (*p* < 0.001)]. The IL-6 gene expression level showed a significant increase and peaked at 0.68 ± 0.09 copies/actin after 12 hours of the U937 cells differentiation (Figure 5C). Decrease in the IL-6 gene expression level was observed to be 0.10 ± 0.01 copies/actin, 0.23 ± 0.02 copies/actin (*p* < 0.01), 0.37 ± 0.04 copies/actin (*p* < 0.001) and 0.45 ± 0.05 copies/actin(*p* < 0.001) at 3, 6, 9 and 12 hours, respectively when the U937 cells during differentiation were treated with 10 μg/ml MeOHGCM6 extract. Inhibition of the IL-6 gene expression levels in the presence of the MeOHGCM6 extract was comparable to that of betamethasone.

### Prophylactic treatment effects of MeOHGCM6 extract on the rats gastric contents following EtOH-induced acute gastric mucosal injury

Gastric mucus secretion, gastric juice volume and gastric mucus pH were determined from the gastric content of rat’s stomach pre-treated with MeOHGCM6 extract following EtOH feeding. 2.45 ± 0.25 gm of gastric mucus and 1.75 ± 0.20 ml of gastric juice were recovered from the stomach of rats fed with only the extract diluent (Table 2). 1.54 ± 0.31 gm of gastric mucus and 0.96 ± 0.25 ml of gastric juice and 1.68 ± 0.19 gm of gastric mucus and 0.96 ± 0.16 ml of gastric juice were recovered from rats pre-treated with 250 and 500 mg/kg b.w. of MeOHGCM6 extract, respectively. In comparison, 2.31 ± 0.42 gm of gastric mucus and 1.15 ± 0.36 ml gastric juice was recovered from rats pre-treated with 20 mg/kg b.w OMP. 2.75 ± 0.49 gm of gastric mucus and 1.75 ± 0.28 ml of gastric juice were recovered from rats fed with similarly prepared unrelated plant extract that served as the NSPE control. This group of control produced high amount of gastric mucus and gastric juice significantly compared to rats treated with the MeOHGCM6 extract. The pH of gastric mucus of rats pre-treated with 2.5, 5.0, 250 and 500 mg/kg b.w. MeOHGCM6 extract were 5.51 ± 0.86, 5.42 ± 0.71, 4.74 ± 0.38 and 3.82 ± 0.24, respectively (Table 2). In comparison, the pH of mucus of rats treated with the extract diluent, 20 mg/kg b.w. OMP and also NPSE control were 7.40 ± 0.15, 6.97 ± 0.37 and 4.35 ± 0.19, respectively.

**Table 2:** The effects of MeOHGCM6 extract pre-treatment on the rat’s gastric contents following EtOH-induced acute gastric mucosal injury

**Pretreatment (*n*** **= 6)**	**Dose (mg/kg b.w.)**	**Mucus production (g)**	**Volume of gastric juice (ml)**	**Gastric pH**
Diluent	-	2.45 ± 0.61	1.75 ± 0.50	7.40 ± 0.37
NSPE	500.0	2.75 ± 1.21	1.75 ± 0.69	4.35 ± 0.47*
OMP	20.0	2.31 ± 1.04	1.15 ± 0.88	6.97 ± 0.92
MeOHGCM6	2.5	2.33 ± 0.77	1.50 ± 0.61	5.51 ± 2.10
	5.0	3.24 ± 1.39	1.50 ± 0.61	5.42 ± 1.73
	250.0	1.54 ± 0.77	0.96 ± 0.62	4.74 ± 0.93*
	500.0	1.68 ± 0.47	0.96 ± 0.40	3.82 ± 0.58*

Rats were fed with the diluent, NSPE, OMP and the different MeOHGCM6 extract concentrations 30 minutes prior to feeding with absolute EtOH. Rats were sacrificed after an hour and gastric mucus secretion was collected and quantified. Results are expressed as mean ± S.D. from six animals.

* Significance difference in comparison to the group pre-treated with 10% Tween 20 (untreated control) value: *p* < 0.05.

### Effects of prophylactic treatment with MeOHGCM6 extract pre-treatment on production of gastric NP-SH following EtOH-induced acute gastric mucosal injury

The level of NP-SH in the stomach was determined from the glandular of rats pre-treated with MeOHGCM6 extract following EtOH-induced acute gastric mucosal injury from the rat’s stomach. The level of NP-SH was at 0.15 ± 0.01 nmol/gm tissue in rats that were fed with the diluent (Table 3). Comparable level (0.15 ± 0.01 nmol/gm tissue) was obtained in stomach of rats pre-treated with 2.5 mg/kg b.w. MeOHGCM6 extract. The levels of NP-SH in groups of rats pre-treated with 5.0, 250 and 500 mg/kg b.w. MeOHGCM6 extract were 0.23 ± 0.01 nmol/g tissue, 0.33 ± 0.03 nmol/g tissue and 0.41 ± 0.02 nmol/gm tissue, respectively and this showed a significant dose-dependent increase. The level of NP-SH in the tissue of the group of rats pre-treated with OMP was 0.33 ± 0.03 nmol/gm and it was comparable to its counterpart in rats pre-treated with 250 mg/kg b.w. MeOHGCM6 extract.

**Table 3:** The effects of MeOHGCM6 extract pre-treatment on the production of gastric NP-SH in EtOH-induced acute gastric mucosal injury

**Pretreatment (*n*** **= 6)**	**Dose (mg/kg b.w.)**	**NP-SH concentration (nmol/g tissue)**
Diluent	-	0.15 ± 0.04
NSPE	500.0	0.23 ± 0.03
OMP	20.0	0.33 ± 0.83*
MeOHGCM6	2.5	0.15 ± 0.57
	5.0	0.23 ± 0.28
	250.0	0.33 ± 0.69*
	500.0	0.41 ± 0.53*

Rats were fed with the different concentrations of MeOHGCM6 extract 30 minutes prior to feeding with absolute EtOH. Rats were sacrificed an hour later and the stomach was rapidly removed, the glandular stomach was collected and the production of NP-SH was determined. Results are expressed as mean ± S.D. from six animals.

* Significance difference in comparison to the group pre-treated with 10% Tween 20 (untreated control) value: *p* < 0.05.

### Effects of MeOHGCM6 extract treatment on gastric lesions following EtOH-induced acute gastric mucosal injury

**Figure 6:**
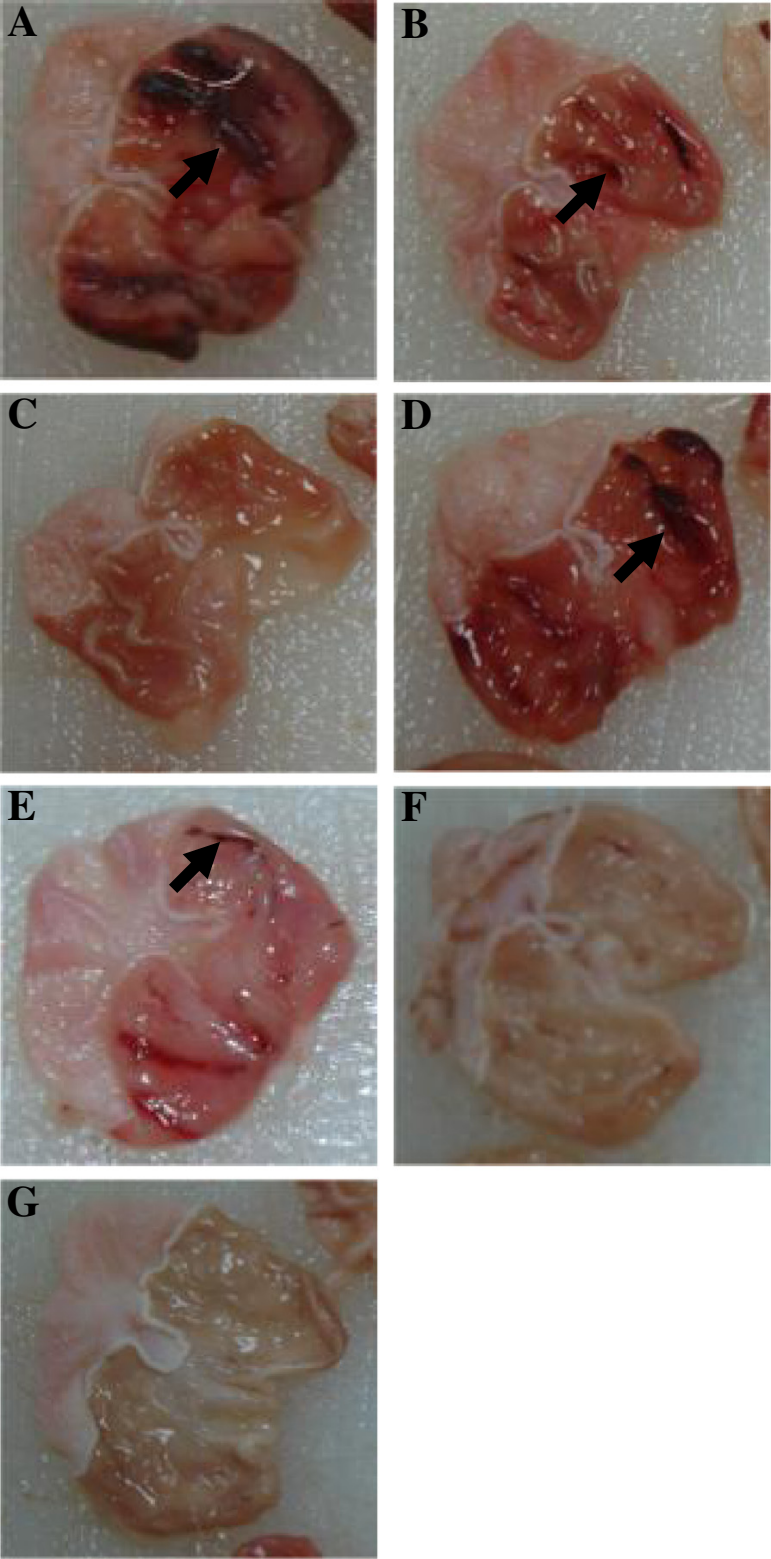
**Effects of MeOHGCM6 extract treatment on the gastric lesions following EtOH-induced acute gastric mucosal injury.** Rats were fed with the different concentrations of MeOHGCM6 extract 30 minutes prior to feeding with 1 ml absolute EtOH. Rats were sacrificed an hour later and the stomach was rapidly removed and incised along the greater curvature. (**A**) Diluent. (**B**) NSPE. (**C**) OMP. (**D**) 2.5 mg/kg b.w. MeOHGCM6. (**E**) 5 mg/kg b.w. MeOHGCM6. (**F**) 250 mg/kg b.w. MeOHGCM6. (**G**) 500 mg/kg b.w. MeOHGCM6. The untreated control stomachs showed extensive EtOH-induced mucosal necrotic lesions mostly in the corpus (arrows). Cells were photographed using a Nikon D70 camera. One archetypal set of photographs are shown.

The gastric histological lesions were observed from the rat’s stomach pre-treated with MeOHGCM6 extract following EtOH-induced acute gastric mucosal injury. Macroscopic examination of the stomach of rats treated with EtOH showed well-defined focal haemorrhagic ulcerations or well-defined hemorrhagic erosions with different sizes of red lines along the long axis of the glandular portion of the stomach (Figure 6A). The major EtOH-induced gastric lesions were approximately about 1290.13 ± 88.16 mm^2^ in size and were observed in rats pre-treated with the extract diluents (Table 4). These findings were consistent with previous reported findings [31]. Reduction in the ulcer area by 675 ± 304.02 mm^2^, 298.93 ± 56.06 mm^2^, 52.80 ± 20.25 mm^2^ and 7.20 ± 3.72 mm^2^ were observed in groups of rats pre-treated with 2.5, 5.0, 250 and 500 mg/kg b.w. MeOHGCM6 extract, respectively, compared to the ulcer area within the stomach of the diluent-treated control group (1290.13 ± 88.16 mm^2^) (Table 4). Almost no lesion (>98%) was noted in rats pre-treated with OMP prior to the treatment with EtOH (Figure 6C). The rats pre-treated with 500 mg/kg b.w. MeOHGCM6 extract showed almost complete (>99%, p < 0.05) inhibition of EtOH-induced ulceration comparable to or better than the findings in rats treated with 20 gm/kg b.w. OMP (Figure 6G, Table 4).

**Table 4:** The effects of MeOHGCM6 extract pre-treatment on the size of the EtOH-induced gastric lesions

**Treatment (*n*** **= 6)**	**Dose (mg/kg b.w.)**	**UA (mm**^**2**^**)**	**Inhibition (%)**
Diluent	-	1290.13 ± 215.95	-
NSPE	500.0	664.27 ± 143.01*	48.51
OMP	20.0	17.47 ± 14.20*	98.65
MeOHGCM6	2.5	675.00 ± 744.69*	47.68
	5.0	298.93 ± 137.31*	76.83
	250.0	52.80 ± 49.61*	95.91
	500.0	7.20 ± 9.11*	99.44

Rats were fed with the different concentrations of MeOHGCM6 extract 30 minutes prior to feeding with absolute EtOH. Rats were sacrificed an hour later and the stomach was rapidly removed and incised along the greater curvature and examined under a dissecting microscope. Results are expressed as mean ± S.D. from six animals.

* Significance difference in comparison to the group pre-treated with 10% Tween 20 (untreated control) value: *p* < 0.05.

## Discussion

Inflammation is a major feature of many diseases. It affects 5% to 7% of the overall population worldwide [32]. Inflammation that occurs in the gastrointestinal tract is by and large causes gastrointestinal ulcer. Gastrointestinal ulcer disease poses a serious economic burden as treatment for the ulcers account to at least 10% of the total cost of treating digestive disorders [33,34]. Effective treatment of this inflammation disorders using safer and effective therapeutics remains an elusive goal. We investigated the standardised MeOHGCM6 extract of a Malaysian red algae, *Gracilaria changii* (Xia et Abbott) Abbott, Zhang et Xia, for its potential anti-inflammatory, gastroprotective and anti-ulcerogenic activities.

In the present study, preparation of the extract was standardized to ensure reproducibility of results and each batch of extract was profiled using LC/MS. The profiling of the MeOHGCM6 extract using LC/MS provided a detailed chromatographic profile of the extract constituents. Highly reproducible MeOHGCM6 extract mass spectral peak profiles were used to mark the fraction identified here as the standardized MeOHGCM6 extract for all the subsequent experimentation. The preliminary profiling of MeOHGCM6 extract revealed the presence of two possible chlorophylls, methyl 10-hydroxyphaeophorbide *a* and 10-hydroxypheophytin as the main constituents of the MeOHGCM6 extract. Consistent with our finding, pheophytin *a,* a type of chlorophyll-related compound that has been found in green algae is proven to have anti-inflammatory activities [35]. Whether these two chlorophylls, contributed to the anti-inflammatory effects described herein is not known and will require further investigation. However, a detailed profiling of standardized MeOHGCM6 extract is required to characterize the compound that was responsible for the mechanism involved.

Inflammatory model using monocyte differentiation to macrophage-like cells was established in the study. Characterization of macrophage-like cells to indicate inflammatory effects is important, since these cells play important role in inflammatory and immune responses and also responsible for various immune functions such as secretion of inflammatory mediators. U937 cell is a suitable model since differentiation of this precursor cell into macrophage-like cells can be studied conveniently in response to various stimuli [36]. Differentiation was determined by cell morphology and by physiological markers. The number of differentiated cell increased when U937 cells were induced to differentiate with increasing concentration of PMA (1–50 nM) and longer incubation period (24–48 hours). In our study, we used viable, intact and metabolically active differentiated U937 cells. During differentiation of the U937 cells, cells were scored for the percentage of viable cells. A dose-dependent reduction in cell viability of the differentiated U937 cells was observed when the cells was induced to differentiate with increasing concentration of PMA (1–50 nM) and longer incubation period (24–48 hours). Based on these results, U937 cells were induced to differentiate with 10 nM PMA for 24 hours in all the subsequent experiments. This study conditions were chosen to ensure monocytic cells were more than 50% differentiated to macrophage-like cells without affecting cell viability and metabolic activity.

The U937 cells during differentiation (macrophage-mediated inflammatory phenomena) were then used to investigate the anti-inflammatory activities of MeOHGCM6 extract. Results presented in this study suggest that MeOHGCM6 extract can exert significant anti-inflammatory effects on TNF-α response level. Reduction in the TNF-α response level was comparable to betamethasone, a commercially available anti-inflammatory drug commonly used as treatment for inflammation [37]. Within 0–8 hours following differentiation of U937 cells, we found a rapid increasing in TNF-α response level. However, the TNF-α response of U937 cells during differentiation was significantly blocked in the presence of the 10 μg/ml MeOHGCM6 extract. Our findings also suggest that MeOHGCM6 extract can exert significant anti-inflammatory effects on TNF-α and IL-6 gene expression level. It was demonstrated by the ability of MeOHGCM6 extract to suppressed TNF-α and IL-6 gene expression level during differentiation of the U937 cells. The suppressive effects of MeOHGCM6 extract on pro-inflammatory cytokines were unlikely to be due to non-specific cell inhibition activity, since 10 μg/ml used for this studies were non-cytotoxic to the cells. This finding is similar to the previous studies which showed that red algae possess the ability to inhibit *in vitro* inflammation and suppress the production of inflammatory mediators [38,39].

Our results also suggest that the MeOHGCM6 extract possesses gastroprotective and anti-ulcerogenic activities. This was confirmed by the ability of MeOHGCM6 extract treatment to ameliorate absolute EtOH-induced acute gastric mucosal injury. Our findings showed that the MeOHGCM6 extract when used to treat rats prior to EtOH feeding accord protection of the gastrointestinal tract against the EtOH-induced acute gastric mucosal injury. The gastroprotective effect of MeOHGCM6 extract was dose-dependent and the effect of treatment with 500 mg/kg b.w. was comparable to OMP, a widely prescribed proton pump inhibitor that inhibits gastric acid secretion used for the treatment of gastric ulcer [40]. The non-specific beneficial effects of any plant extract is not possible since we showed that rats pre-treated with an unrelated but similarly prepared plant extract (NSPE), did not present similar gastric responses and substantial inhibition of the EtOH-induced acute gastric mucosal injury. The mechanism contributing to the gastroprotective effects of MeOHGCM6 extract, however is likely to be different from that of OMP. The extract reduced gastric juice and gastric mucus secretion and lowered the stomach juice pH to 4.74 ± 0.38 at 250 mg/kg b.w. In contrast, OMP reduced the gastric mucus secretion and neutralized stomach juice pH to 6.97 ± 0.37. Our findings from OMP treatment are similar to that previously reported [41]. Neutralization of stomach pH is among the known side effects of OMP which could contribute to the alteration in the stomach normal flora thus, predisposing to diarrheal diseases. Decreasing the stomach juice pH by MeOHGCM6 extract could be useful for restoration of the normal flora [42] which might have contributed to the gastroprotective and anti-ulcerogenic property of the MeOHGCM6 extract. Our results also suggest that MeOHGCM6 extract similar to OMP increases the gastric NP-SH content. NP-SH has been shown to accord gastroprotective effects against oxygen radicals inducing agents such as EtOH [43,44]. The mechanisms of EtOH-induced acute gastric mucosal injury are thought to arise as a result of depletion of gastric mucus content, damaged mucosal blood flow, haemorrhage and necrotic of mucosal tissues and over production of free radicals which lead to an increased lipid peroxidation [45]. The oxygen radicals will then initiated the oxidative stress which contributes to all forms of gastrointestinal ulcer. Consistent with our finding, the red algae, *Gracilaria changii* has previously been reported to possess free radical scavenging activity [46]. A recent study has also shown that algae have anti-oxidant properties which could be beneficial in the prevention or treatment of gastric ulcers [22,47-49].

## Conclusion

We demonstrated that the LC/MS standardised MeOHGCM6 extract could modulate macrophage-mediated pro-inflammatory cytokines over-production *in vitro*. This property is consistent with its ability to accord gastro protective effects against the EtOH-induced acute gastric mucosal injury and ameliorate the healing of the ulcers. The gastroprotective potentials of MeOHGCM6 extract may be in part due to its ability to act as and reduced stomach acidity. An algae-based medication with anti-inflammatory and gastric anti-ulcerogenic activity is of great therapeutic importance as most of the anti-inflammatory drugs used in modern medicine are ulcerogenic. Further studies, however, are required for elucidating the exact molecular and cellular mechanisms of action and the effects of MeOHGCM6 extract in the treatment of chronic ulcers.

## Competing interests

The authors declare that they have no competing interests.

## Authors’ contributions

MHS performed the majority of the experiments, analyzed the data and wrote the manuscript; DA performed some parts of the experiment, provided part of the reagents/analytical tools and analyzed the data; KZ wrote the manuscript; SAB designed the experiment, provided the reagents/analytical tools, analyzed the data and wrote the manuscript. All authors read and approved the final manuscript.

## Pre-publication history

The pre-publication history for this paper can be accessed here:

http://www.biomedcentral.com/1472-6882/13/61/prepub
